# Strategies to facilitate the discovery of novel CNS PET ligands

**DOI:** 10.1186/s41181-016-0016-2

**Published:** 2016-09-13

**Authors:** Lei Zhang, Anabella Villalobos

**Affiliations:** Neuroscience and Pain Medicinal Chemistry, Pfizer Worldwide Research and Development, 610 Main Street, Cambridge, MA 02139 USA

**Keywords:** PET, Positron emission tomography, CNS, CNS PET MPO, Cold tracer method, Radioligand discovery

## Abstract

Positron Emission Tomography (PET), as a non-invasive translatable imaging technology, can be incorporated into various stages of the CNS drug discovery process to provide valuable information for key preclinical and clinical decision-making. Novel CNS PET ligand discovery efforts in the industry setting, however, are facing unique challenges associated with lead design and prioritization, and budget constraints. In this review, three strategies aiming toward improving the central nervous system (CNS) PET ligand discovery process are described: first, early determination of receptor density (B_max_) and bio-distribution to inform PET viability and resource allocation; second, rational design and design prioritization guided by CNS PET design parameters; finally, a cost-effective in vivo specific binding assessment using a liquid chromatography-mass spectrometry (LC-MS/MS) “cold tracer” method. Implementation of these strategies allowed a more focused and rational CNS PET ligand discovery effort to identify high quality PET ligands for neuroimaging.

## Review

**Fig. 1:**
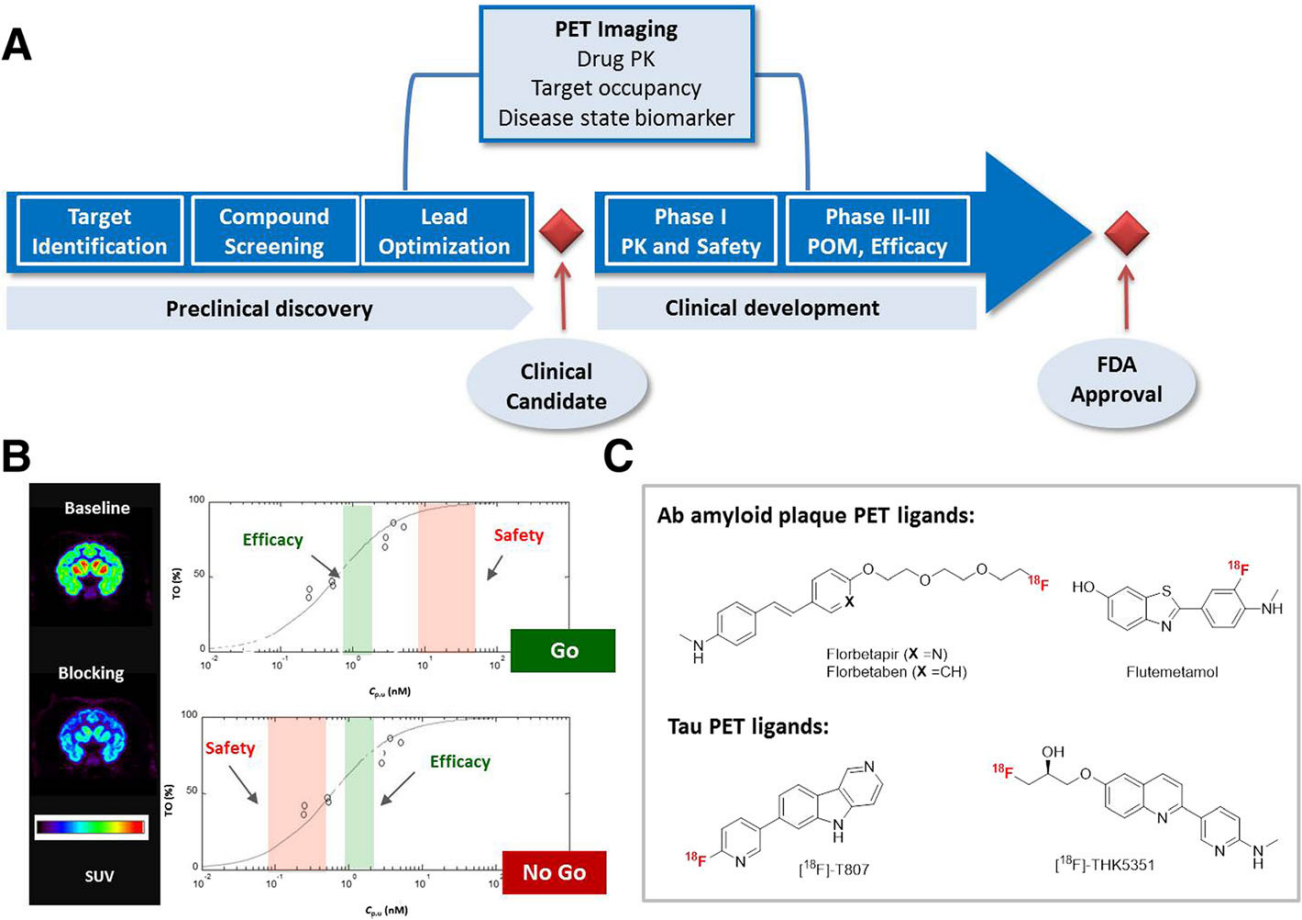
Applications of PET imaging in the CNS drug discovery process

Central nervous system (CNS) disorders, such as Alzheimer’s disease (AD), schizophrenia and Parkinson disease (PD), are among some of the most debilitating illnesses wherein significant unmet medical needs exist. CNS drug discovery faces significant challenges due to the intrinsic complexity of the human brain and scarcity of predictive preclinical efficacy models and disease-relevant biomarkers (Manji & DeSouza 2008). Positron emission tomography (PET), non-invasive in nature, can play a multi-faceted role in the CNS drug discovery process to directly address some of the key challenges (Fig. 1a) (Lee & Farde 2006). With a radiolabeled drug molecule, PET imaging can be used to quantify the drug molecule’s bio-distribution (Fischman et al. 2002) and provide unequivocal neuro-pharmacokinetic (neuroPK) endpoints, such as brain permeability, that otherwise can’t be directly measured. In addition, PET imaging can be used to quantify occupancy of a drug molecule to the specific biological target of interest (Grimwood & Hartig 2009). Target occupancy results, in turn, can be used to inform critical decisions at different stages of the drug discovery process. For example, target occupancy, together with efficacy and safety endpoints, can be used to differentiate pre-clinical leads and select the best molecule to advance to clinical trials (Fig. 1b) (Matthews et al. 2012). Clinically, target occupancy can be used to unequivocally confirm “drug-on-target” (proof of mechanism), (Morgan et al. 2012) guide clinical dose selections, and enable data-driven clinical “Go/No Go” decisions depending on whether the onset of side effects occurs prior to or *after* desired target occupancy range for efficacy (Wong et al. 2009). Finally, PET imaging, with ligands specific to targets that are associated with a given disease state, can be used as disease state biomarkers for early disease detection, disease progression monitoring and patient phenotyping. Prominent examples are FDA-approved Aβ plaque PET ligands (Ono & Saji 2015) [e.g. florbetapir, (Eli Lilly Pharmaceuticals Press Release 2012) florbetaben (Piramal Imaging 2014) and flutemetamol (GE Healthcare Press Release 2013)] and emerging Tau PET ligands (Choe & Lee 2015) [e.g. [^18^F]T-807 (Xia et al. 2013) and [^18^F]THK-5351 (Harada et al. 2016)], which detect Aβ amyloid plaque and neurofibrillary tangles accumulation in brain, two main hallmarks of AD pathology (Fig. 1c).

To enable PET imaging, it is necessary to develop high quality target-specific CNS PET ligands that meet a distinctive set of criteria (Table 1). Structurally, a CNS PET ligand needs to contain a structural moiety amenable for [^11^C] or [^18^F] incorporation. Considering the short half-lives of PET radionuclides (20 min for [^11^C] and 110 min for [^18^F]), late-stage radiolabel incorporation is necessary to allow rapid synthesis and purification. With regard to pharmacology, a CNS PET ligand should be potent (B_max_/K_d_ >10, see section on ‘understand the target’) and selective towards the target of interest (typically > 30×) and needs to occupy the same binding pocket as the drug molecule to allow competitive blocking and target occupancy measurement. In terms of pharmacokinetic (PK) properties, a CNS PET ligand must be brain penetrant, but should not form brain-permeable radioactive metabolites which can confound radioactivity measurements. Furthermore, it must demonstrate low non-specific binding (NSB) to brain white matter to achieve sufficient signal-to-noise ratio for quantification. Finally, similar to drug molecules, a CNS PET ligand must demonstrate safety for clinical dosing. Considering low clinical doses of PET ligands (typically < 10 μg), a simplified microdosing good laboratory practice (GLP) toxicity study is required for exploratory investigative new drug (eIND) submission, involving acute intra venous (IV) dosing in a single species, typically rats, plus 14-day observation (Wagner & Langer 2011). Compared to CNS drug molecules, a CNS PET ligand may have more stringent criteria regarding potency (single digit nM or sub-nM), and PK, such as minimal brain permeable radioactive metabolite formation and low NSB.

**Table 1:** Desired attributes for successful CNS PET ligands

Structure requirements	PK properties
➢ Structural handle for ^18^F or ^11^C labeling	➢ Brain permeable
➢ Amenable for late-stage radiolabeling	➢ No brain permeable radioactive metabolites
Pharmacology	➢ Low non-specific binding (NSB)
➢ Occupy the same binding site for the clinical drug candidate	Safety
➢ High potency (typically low single digit nM to sub-nM)	➢ Safe for clinical dosing, typically at μg scale
➢ High selectivity	➢ Microdosing GLP: single species IV + 14 day observation

**Fig. 2:**
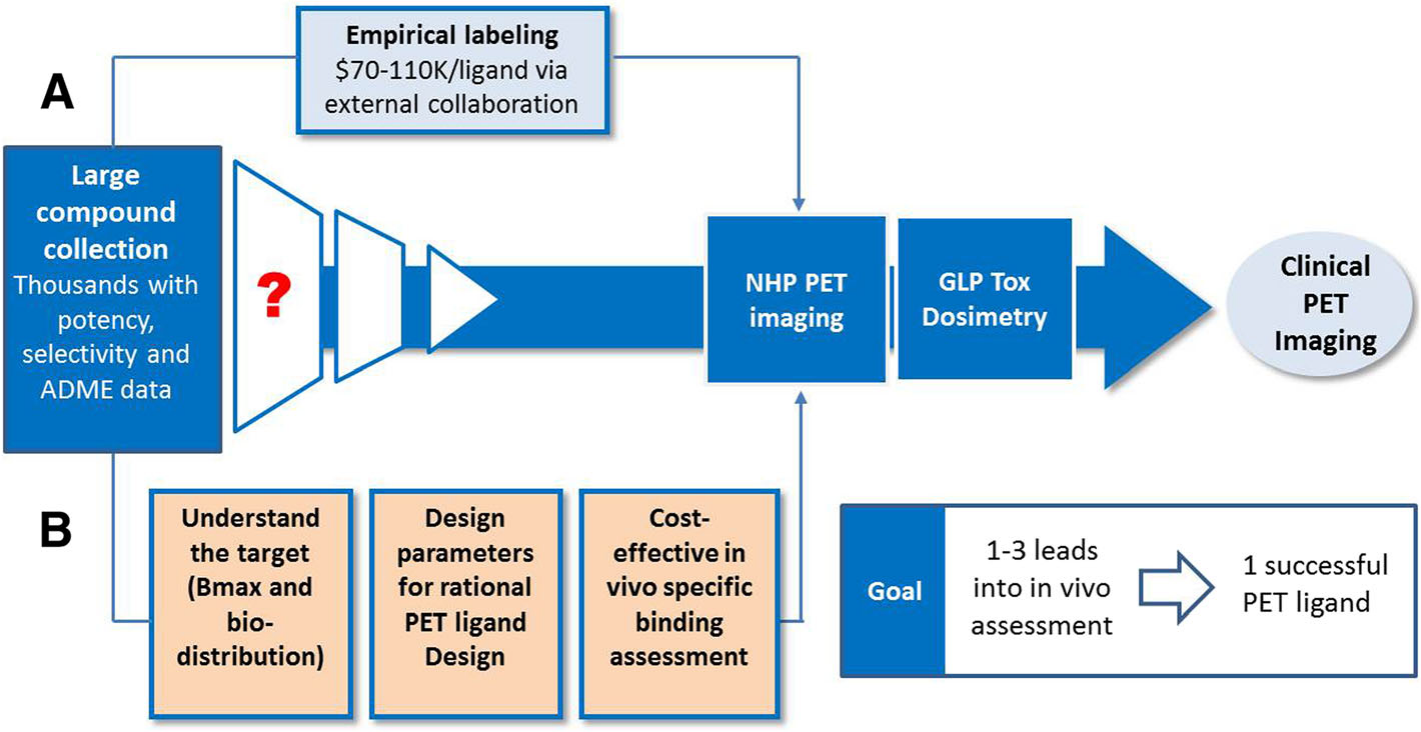
Strategies to further streamline the CNS PET ligand discovery process and improve success rate

Considering the importance of PET imaging in CNS drug discovery, it is crucial to establish an efficient process for CNS PET ligand discovery. Specifically in the industry setting, there are often large collections of existing chemical matter (up to thousands of compounds), from drug discovery efforts and literature, with a wealth of potency, selectivity and in vitro absorption, distribution, metabolism and excretion (ADME) data. While this provides an enviable starting point for PET ligand discovery effort, it also presents a distinctive challenge on how to identify the right leads for PET imaging from such large compound pools. In the past, prioritization was carried out in a largely empirical manner, with a primary focus on potency. A number of radiotracer leads, sometimes more than 10, were radiolabeled and screened in PET imaging to identify one viable PET ligand. This “multiple shots on goal” approach (Fig. 2, path a) was resource intensive and not sustainable, considering the significant cost associated with non-human primate (NHP) PET imaging studies. Therefore, it is imperative to identify new strategies to significantly improve the PET ligand discovery process in a more rational way with higher success rate, lower cost, and less resources. In an ideal state, we envision advancing no more than three ligand candidates, preferably one, into PET imaging studies to yield a successful PET ligand for clinical imaging (Fig. 2).

Herein, three strategies aiming to improve the overall success rate and efficiency of the novel CNS PET ligand discovery process (Fig. 2, Path b) are described. First, “*understand the target*”: we aimed to gain an early understanding of the expression level (B_max_) and bio-distribution of the specific target to determine PET viability and study design. Second, “*design the right molecule*”: we aimed to define a set of tractable design and selection parameters to guide rational PET ligand design. Third, “*implement cost*-*effective* in vivo *model*”: we aimed to explore cost-effective methods to provide an early read on the in vivo specific binding of ligand candidates prior to triggering more resource-extensive non-human primate (NHP) PET imaging studies. It was also critical that all three strategies could be carried out efficiently so that higher success rates would not come at the cost of longer timelines.

### Bmax and bio-distribution: understand the target

To assess PET viability, it is important to have a clear read on two specific parameters regarding the biological target: the maximum concentration of target binding sites (B_max_) and brain bio-distribution. B_max_ represents target expression level and is key to inform the level of affinity (K_d_) required for a successful radiotracer (B_max_/K_d_ ≥ 10) (Patel & Gibson 2008). The lower the expression level, the higher the affinity required for a radiotracer to show in vivo specific binding. If a given target has a B_max_ value less than 1 nM, it will be challenging to identify PET ligand leads with sufficient potency and alignment of other properties (e.g. PK, NSB). Early determination of B_max_ would thus allow teams to assess the likelihood of success of PET strategy for various targets and help allocate resources to more viable targets (e.g. B_max_ > 1 nM). Brain bio-distribution, while not impacting PET doability, is required to inform subsequent PET imaging studies such as specific binding assessment. For example, if a target is only expressed or enriched in certain brain regions [e.g. striatum for phosphodiesterase 10 (PDE10) (Tu et al. 2010)] then a target-free brain region could be used as a “reference region” (e.g. cerebellum for PDE10) to determine specific binding. On the other hand, if a target is expressed throughout the brain [e.g. PDE4, (Pérez-Torres et al. 2000) metabotropic glutamate receptor type 5 (mGluR5) (Romano et al. 1995)], it would be necessary to carry out both baseline and blocking or equivalent (e.g. knock-out animal) studies to determine specific binding. For any novel CNS target that lacks pre-existing knowledge on B_max_ and bio-distribution, we took an approach to quickly identify a suitable [^3^H] or [^125^I] radiotracer to enable saturation binding and in vitro autoradiography studies for B_max_ and bio-distribution determination. Unlike PET ligands, a [^3^H] or [^125^I] radiotracer is not necessarily required to be brain permeable as the studies are carried out in vitro, hence taking much less effort to develop a suitable ligand. Furthermore, a [^3^H] or [^125^I] radiotracer can be used to establish binding assays early on to identify leads with potent binding affinity for PET consideration. Taken together, a small upfront effort in identifying a target-specific [^3^H] or [^125^I] radiotracer could lead to significant time and cost saving down the road and could be considered as pre-requisite to initiating any extensive PET discovery effort for a novel CNS target.

### PET ligand design parameters: design the right molecule

**Fig. 3:**
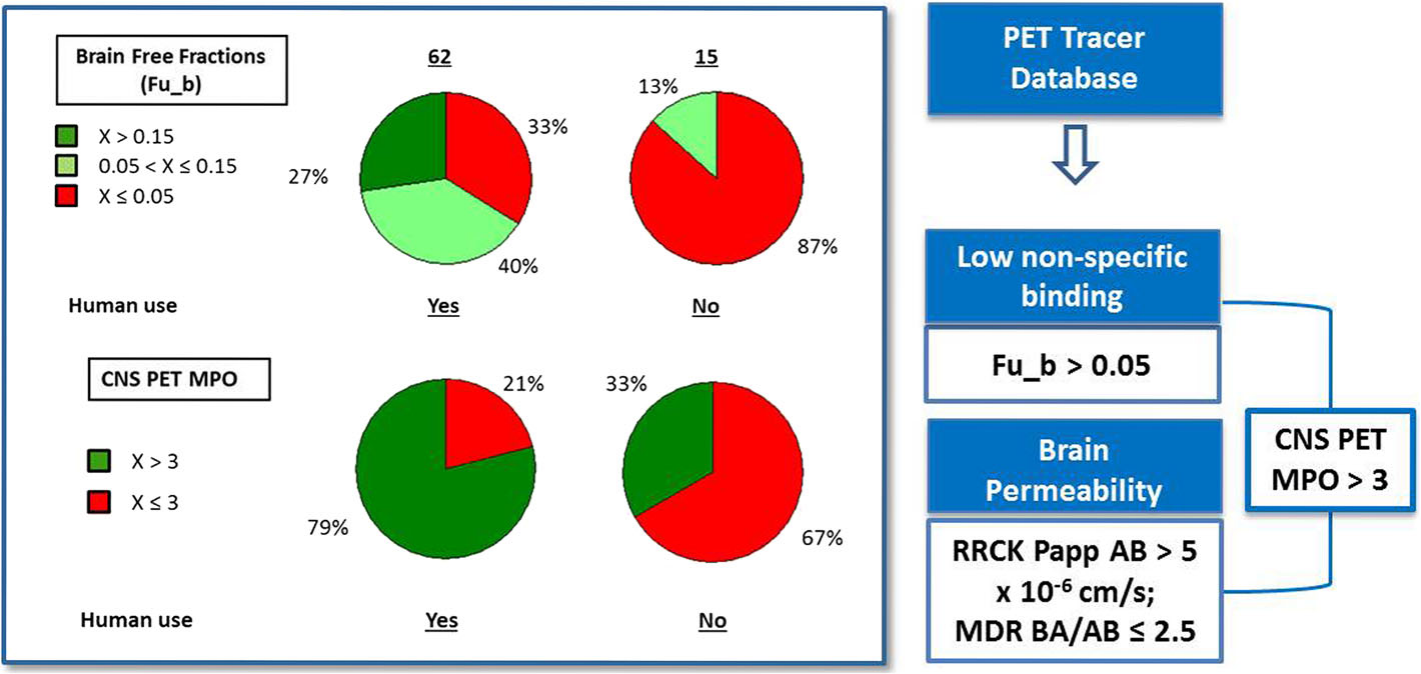
CNS PET ligand design parameters

Effective PET ligand discovery calls for facile prioritizations of best leads from a large pool of existing chemical matter and focused structure-activity relationship (SAR) efforts to rapidly design and identify suitable candidates for PET imaging. In an effort to understand the preferred property space for CNS PET ligands, our group compiled a PET ligand database consisting of 62 clinically validated CNS PET ligands and 15 unsuccessful radioligands as negative controls. A systematic analysis was then carried out, in which key differences between the two categories in terms of physicochemical properties and in vitro ADME properties were identified (Zhang et al. 2013). As shown in Fig. 3, for in vitro ADME properties, fraction unbound in brain (Fu_b) (Di et al. 2011) emerged as a pronounced differentiator across two categories. A Fu_b value of greater than 0.05 should be targeted to minimize the risk of NSB as it captured the majority of the successful ligands (67 %), while only 13 % of the failed ligands were in this range. Passive permeability as measured by Ralph Russ canine kidney (RRCK) apparent permeability coefficient (P_app_) apical-to-basolateral (AB) and efflux risk as measured by multidrug resistance protein 1 (MDR1) basolateral-to-apical/apical-to-basolateral (BA/AB) ratios were identified as two important parameters to predict brain permeability. Specifically, RRCK P_app_ AB values > 5 × 10^−6^ cm/s and MDR1 BA/AB ratios ≤ 2.5 should be targeted to increase the probability of the ligand leads getting into the brain. For the analysis of physicochemical properties, we used a novel tool, CNS PET multi-parameter optimization (MPO) (scores ranging from 0 to 6). This multi-parameter optimization tool allowed us to track all six physicochemical properties commonly considered in compound design, including cLogP, cLogD, MW (molecular weight), tPSA (topological polar surface area), HBD (number of hydrogen bond donors), and pKa (ionization constant of the most basic center). Compared to individual physicochemical property parameters, CNS PET MPO provided much improved differentiations between the two categories, with CNS PET MPO > 3 preferred. Only a low percentage (33 %) of the failed ligands and the vast majority (79 %) of the successful ligands resided in this range. Further analysis also revealed that by targeting CNS PET MPO > 3, there was a higher probability to align all three key ADME parameters in one molecule. Importantly, our group has developed high performing in silico models for RRCK, MDR and Fu_b based on experimental data of a diverse set of >100,000 compounds, which allows calculations of in vitro ADME properties for assessment prior to synthesis. These in vitro ADME and physicochemical property criteria, together with previously described potency criteria (B_max_/Kd > 10), should be considered for lead prioritization and rational ligand design.

**Fig. 4:**
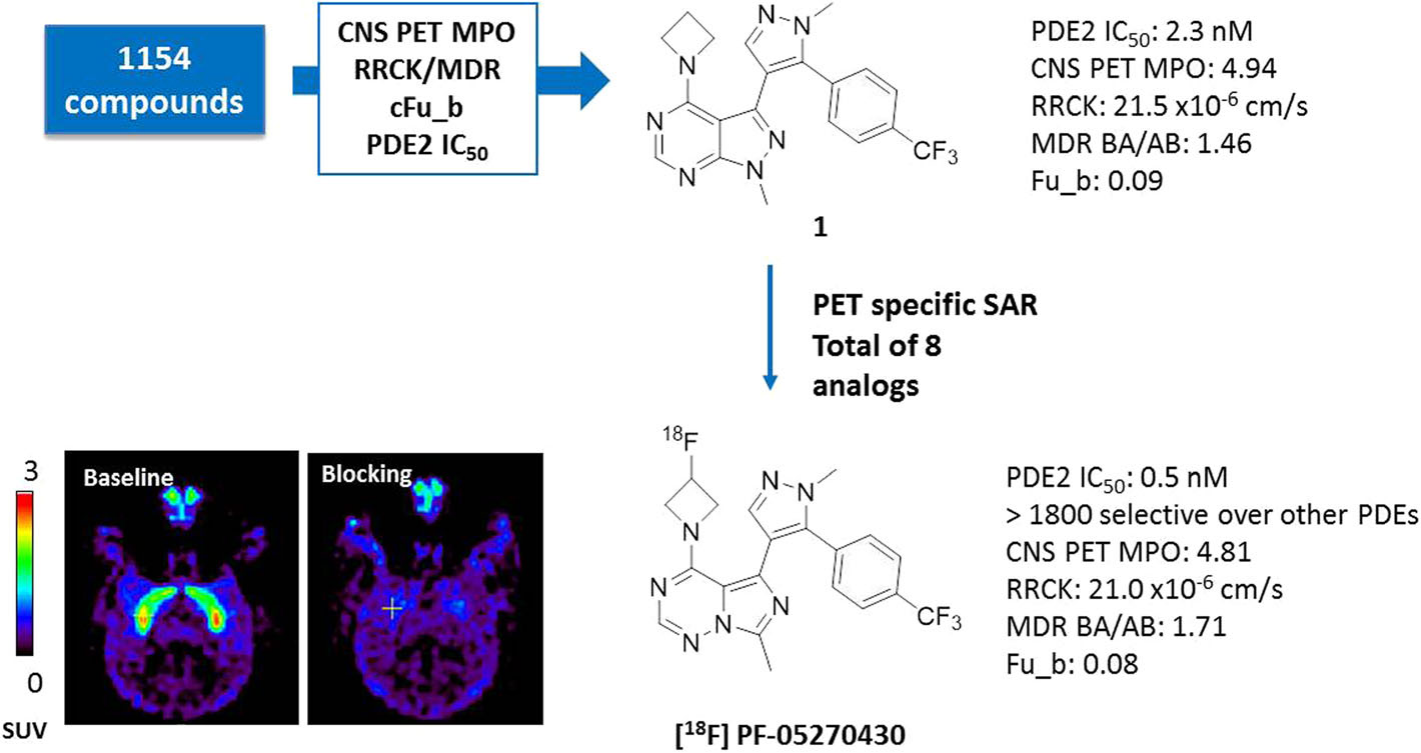
Discovery of a PDF_2A_-selective PET ligand [^8^F]PF-05270430 guided by CNS PET ligand design parameters

The prospective use of these PET design parameters was exemplified by the development of a highly selective PDE_2A_ PET ligand 4-(3-[^18^F]fluoroazetidin-1-yl)-7-methyl-5-(1-methyl-5-(4-(trifluoromethyl)phenyl)-1*H*-pyrazol-4-yl)imidazo[1,5-*f*] [1,2,4] triazine ([^18^F]PF-05270430) (Zhang et al. 2013). As illustrated in Fig. 3, starting from over one thousand existing chemical compounds, upon filtering by the defined criteria of CNS PET MPO, RRCK/MDR, and Fu_b, and further prioritization by potency, we quickly identified pyrazolopyrimidine **1** as a promising PET ligand lead. PET assessment of [^11^C]**1** in NHP revealed preferential binding to striatum, consistent with PDE_2A_ enzyme expression. However, in vivo binding potential (BP) of 0.6 was sub-optimal for practical utility in receptor occupancy quantification. To address this issue, we carried out a PET-specific structure activity relationship (SAR) effort with the goal of further improving potency while maintain other properties. A total of six close-in analogs were targeted for synthesis, with calculated properties in the desired ranges. From this cohort, imidazotrazine PF-05270430 was identified as the best lead to advance for PET assessment (Fig. 4). Compared to compound **1**, PF-05270430 achieved ~4× improvement in potency (IC_50_ = 0.5 nM) without compromising selectivity and other properties. As predicted by the in silico models, PF-05270430 fit our defined preferred PET parameters nicely, including high CNS PET MPO (4.86), high RRCK P_app_ AB (21.0 × 10^−6^ cm/s) and low MDR BA/AB (1.71) suggesting good brain permeability and favorable Fu_b (0.08) with a low risk of non-specific binding. In addition, the fluorine on the terminal azetidine provided a synthetic handle for [^18^F] labeling. In the subsequent PET imaging studies, the improvement in potency translated nicely into higher in vivo BP_ND_ (1.8 in putamen, 1.4 in caudate). Importantly, the signal of [^18^F]PF-05270430 in striatum was significantly blocked by a selective PDE_2A_ inhibitor PF-05180999 in a dose-responsive manner, confirming its in vivo specificity for the PDE_2A_ enzyme. These results suggested that [^18^F]PF-05270430 could be used as a promising ligand for PDE_2A_ PET imaging.

**Fig. 5:**
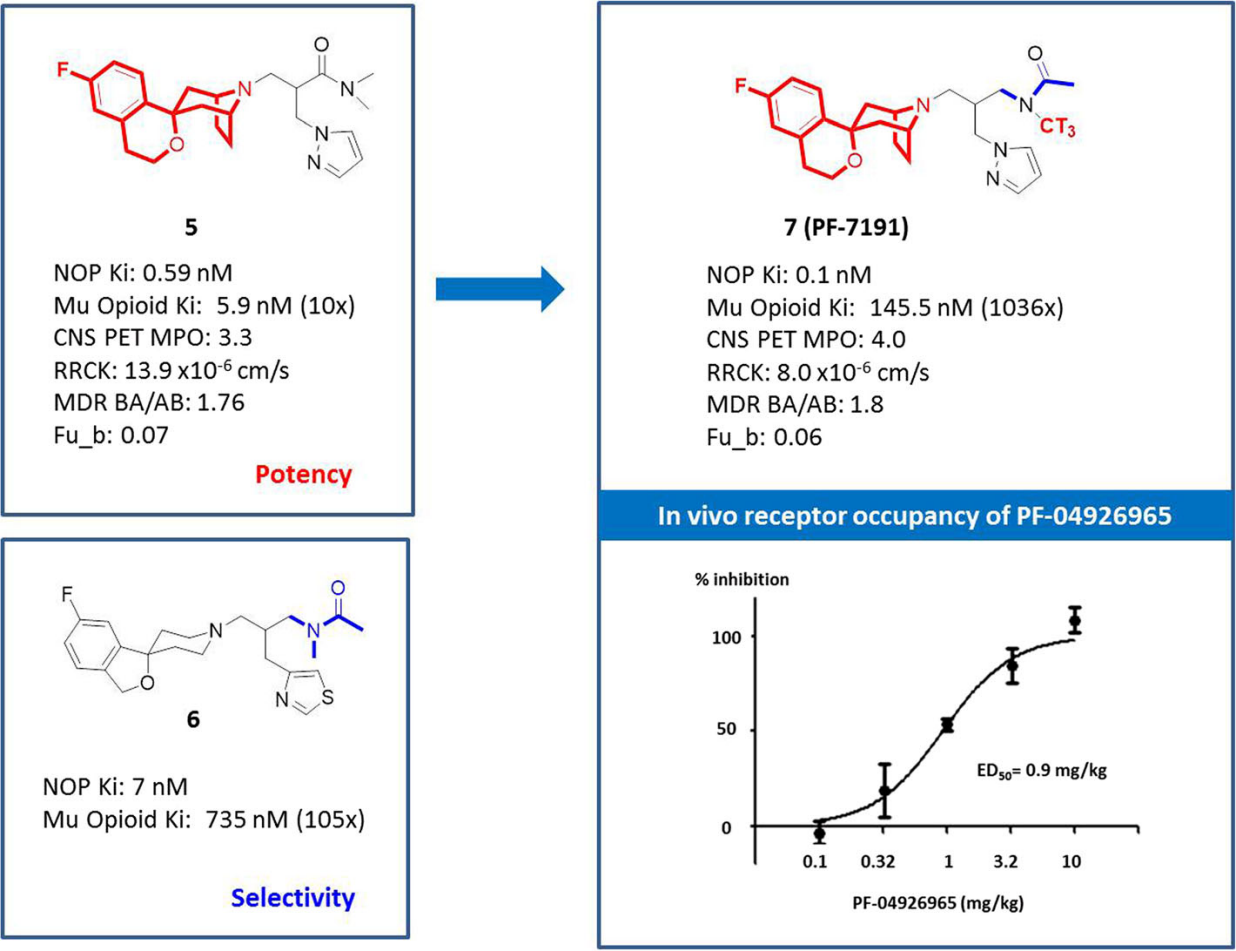
Discovery of a NOP-selective in vivo radiotracer [^3^H]PF-7191 guided by CNS PET ligand design parameters

The PET design parameters have also been applied to the discovery of tritiated in vivo radiotracers. This was illustrated by our effort in the discovery of a nociceptine opioid receptor (NOP) in vivo radiotracer, (+)-*N*-(2-((1*H*-pyrazol-1-yl)methyl)-3-((1*R*,3*r*,5*S*)-6'-fluoro-8-azaspiro[bicyclo[3.2.1]octane-3,1'-isochroman]-8-yl)propyl)-*N*-[^3^H]-methylacetamide ([^3^H]PF-7191) (Fig. 5) (Zhang et al. 2014). Past efforts in the NOP radiotracer discovery were unsuccessful largely due to high in vivo NSB, likely a result of the highly lipophilic nature of previous ligand leads. To identify better tracer leads, we started off by mining the data for existing chemical matter, prioritized by the CNS PET design parameters. From this effort, a spirocyclic compound **5** caught our attention, as it demonstrated desired sub-nM affinity to the NOP receptor (*K*_i_ = 0.59 nM) with well-aligned properties including CNS PET MPO of 3.3 (>3), RRCK P_app_ AB of 13.9 × 10^−6^ cm/s (>5), MDR BA/AB of 1.76 (≤2.5) and Fu_b of 0.07 (>0.05). However, it lacked sufficient selectivity over mu-opioid receptor (10×). Further in-depth SAR analysis revealed that mu-opioid receptor selectivity could be modulated by reversing the amide connectivity at the right hand *N*-alkyl portion, as exemplified by compound **6** (105× selectivity). Combining structure features of compound **5** with the reversed amide selectivity handle from compound **6** yielded a much improved radiotracer lead PF-7191. As shown in Fig. 4, PF-7191 maintained all desired CNS PET properties with significantly improved potency (*K*_i_ = 0.1 nM) and selectivity over mu-opioid receptor (1036×). The bio-distribution of [^3^H]PF-7191 in rat brain was determined through an ex vivo binding study. Four brain regions (cortex, hippocampus, striatum and cerebellum) were examined. The NSB was determined using a high dose of a selective NOP receptor antagonist (PF-04926965, 10 μM). As shown in Fig. 3, the distribution of [^3^H]PF-7191 binding was consistent with known NOP receptor expression: high in cortex and hippocampus and low in striatum and cerebellum. Robust specific binding was observed in all four brain regions tested with cortex showing the highest specific binding with a specific/non-specific binding ratio of ~18. In the subsequent in vivo receptor occupancy study, [^3^H]PF-7191 demonstrated rapid brain uptake and a high percentage (~80 %) of the total binding in rat cortex was determined to be receptor specific. The binding of [^3^H]PF-7191 was inhibited by PF-04926965, a selective NOP receptor antagonist, in a dose–response manner, yielding an ED_50_ of 0.9 mg/kg. This overall favorable profile indicated that [^3^H]PF-7191 is a robust radiotracer to support preclinical in vivo receptor occupancy measurements and a promising lead for C-11 labeling and further PET assessment in higher species.

### LC-MS/MS “cold tracer” method: implement cost-effective in vivo specific binding assessment

In vivo specific binding assessment studies are necessary to confirm the viability of a potential PET ligand in preclinical species prior to advancing into human studies. For in vivo specific binding assessment, our earlier efforts employed the traditional in vivo radiotracer target occupancy (TO) protocol in rodents and used it as a pre-screen prior to advancing potential ligand leads to the more costly NHP PET imaging studies. In these efforts, mice were treated with either vehicle (baseline) or a high dose of a blocking compound (blocking), followed by administration of a tritiated version of a PET ligand lead. After tissue dissection and sample preparation, the radioligand binding in brain regions of interest was quantified by scintillation spectroscopy. The specific binding was determined by comparing a vehicle group with a blocking group pre-treated with a high dose of a target-selective compound or a wild-type (WT) group with a target knock-out (KO) group mimicking complete target occupancy. While this method could provide valuable specific binding information, it had significant drawbacks. First of all, there was considerable cost and time associated with precursor preparation and tritiation of suitable brain penetrant ligand leads. Furthermore, the substrate scope was limited as not all ligand leads had sites for tritiation. For example, leads that only had a [^18^F] labeling handle would not be amenable to this method.

**Fig. 6:**
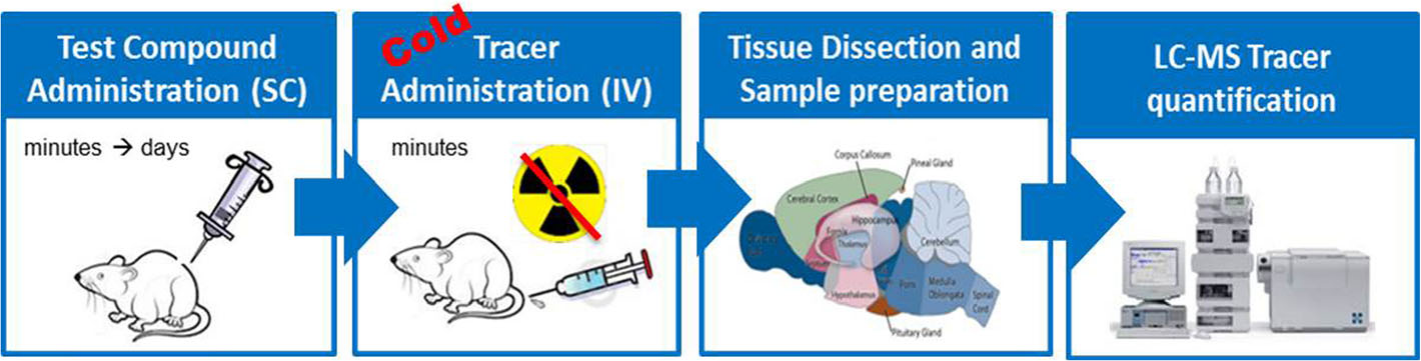
LC-MS/MS “cold tracer” method

To address these limitations, we explored a previously reported liquid chromatography-mass spectrometry (LC-MS/MS) “cold tracer” protocol for in vivo specific binding assessment (Fig. 6) (Chernet et al. 2005). Recent advances in high sensitivity LC-MS/MS have enabled accurate quantification of low compound concentrations at tracer levels. Therefore, rather than administration of a radioactive “hot” ligand, a ligand candidate in a *non*-*radiolabeled* “cold” form at a low tracer dose (typically ≤ 10 μg/kg) was injected into test animals. The distribution of the “cold” tracer in various brain regions was then quantified by high sensitivity LC-MS/MS in place of scintillation spectroscopy. These modifications offered significant improvements over the traditional radioactive TO method in multiple fronts. First, the “cold tracer’ method bypassed the tritiation step, saving time and cost, and importantly this method was applicable to all PET ligand chemotypes. Second, it provided exposure measurements for both the radioligand lead and blocking compound, yielding TO and PK information in a single experiment. Finally, it allowed concurrent use of multiple “cold” tracers in the same animals to enable sophisticated pharmacology studies that would not be possible otherwise. All these advantages mounted to a faster and more cost-effective way to test new PET ligand leads in vivo. It is important to point out, however, that in certain situations wherein there is significant rodent/human species disconnect, either in target expression (B_max_) or binding affinity of a PET ligand lead, then study in rodents would no longer predict PET ligand performance in human. In situations like this, one could consider testing the ligand leads directly in higher species to gain an accurate read on ligand viability.

**Fig. 7:**
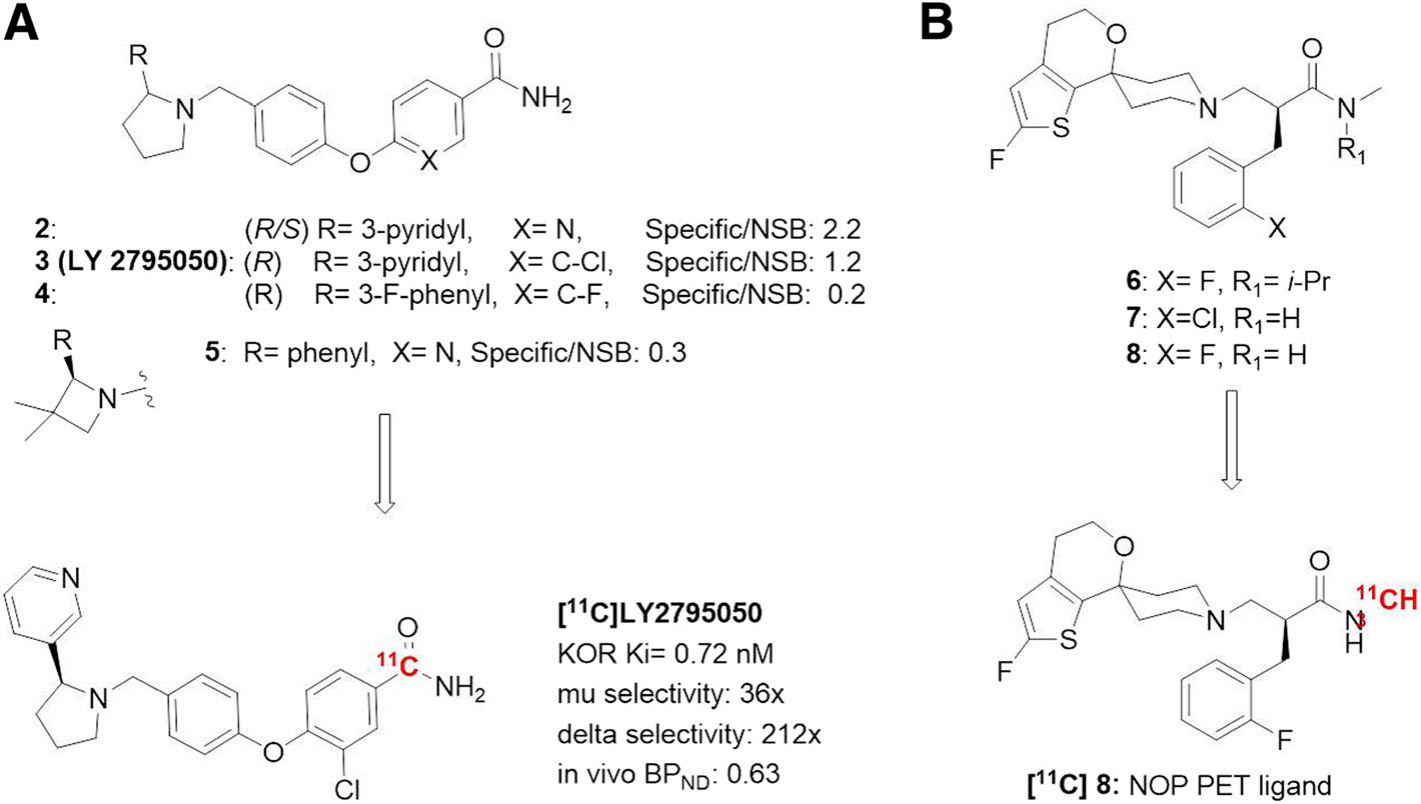
Discovery of novel CNS PET ligands facilitated by the LC-MS/MS method

The usage of LC-MS/MS “cold tracer” method was nicely demonstrated by the discovery of a novel kappa opioid receptor (KOR) antagonist PET ligand [^11^C]-3-chloro-4-[4-[[(2*S*)-2-(pyridine-3-yl)pyrrolidin-1-yl]methyl]phenoxy]benzamide ([^11^C]LY2795050) (Fig. 7a) (Mitch et al. 2011). SAR efforts around a novel aminobenzyloxyarylamide chemical series yielded four KOR antagonists that were selected for in vivo specific binding evaluation in rats by the LC-MS/MS. The level of specific binding was determined by comparing brain uptake in the KOR-enriched striatum region, representing total binding, with the KOR-free cerebellum region, representing NSB. Among four compounds assessed, compound **2** demonstrated highest specific to NSB ratio (2.2). However its brain permeability was poor. Of the remaining three compounds, compound **3** (LY2795050) emerged as the best lead with appropriate brain kinetics and a specific to NSB ratio (1.2) comparable to (−)-4-methoxycarbonyl-2-[(1-pyrrolidinylmethyl]-1-[(3,4-dichlorophenyl)acetyl]-piperidine (GR103545) (1.4), a known KOR agonist PET ligand. The striatal brain uptake of LY2795050 was dose-dependently blocked by a KOR antagonist compound **4** [0.1–30 mg/kg, per oral (PO)]. LY2795050 was also evaluated in WT and KOR-KO mice following the same LC-MS/MS protocol. Consistent with the findings in rats, LY2795050 showed higher concentrations in the KOR-rich striatum region, with a striatum-to-cerebellum ratio of 3.3 at 60 min after dosing. LY2795050 was subsequently radiolabeled by [^11^C] and evaluated in a NHP PET imaging study (Zheng et al. 2013). As predicted by rodent LC-MS/MS “cold tracer” studies, [^11^C]LY2795050 showed favorable metabolic profile and reasonable KOR-specific binding signals. The level of specific binding as measured by *BP*_ND_ was 0.63 in cingulate cortex and 0.66 in putamen.

In another example, the LC-MS/MS “cold tracer” method was used to facilitate the discovery of a novel nociceptin/orphanin FQ peptide (NOP) PET tracer [^11^C]-(*S*)-3-(2’-fluoro-4’,5-dihydrospiro[piperidine-4,7’-thieno-[2,3-*c*]pyran-1-yl)-2(2-fluorobenzyl)-*N*-methylpropanamide (**8**) (Fig. 7b) (Pike et al. 2011). In this effort, three potential ligand leads were tested in rats using the LC-MS/MS cold tracer method. Brain tissue regions corresponding to high NOP and low NOP expression, hypothalamus and striatum respectively, were examined. All three ligand leads demonstrated fast kinetics and high brain uptake. Roughly three-fold higher uptake was observed in NOP-rich hypothalamus than in NOP-poor striatum, suggesting reasonable NOP receptor-specific binding. Subsequent evaluation in NHP PET imaging revealed compound **8** provided best specific binding in NHP with an estimated specific to non-specific ratio of 1.28.

## Conclusions

**Fig. 8:**
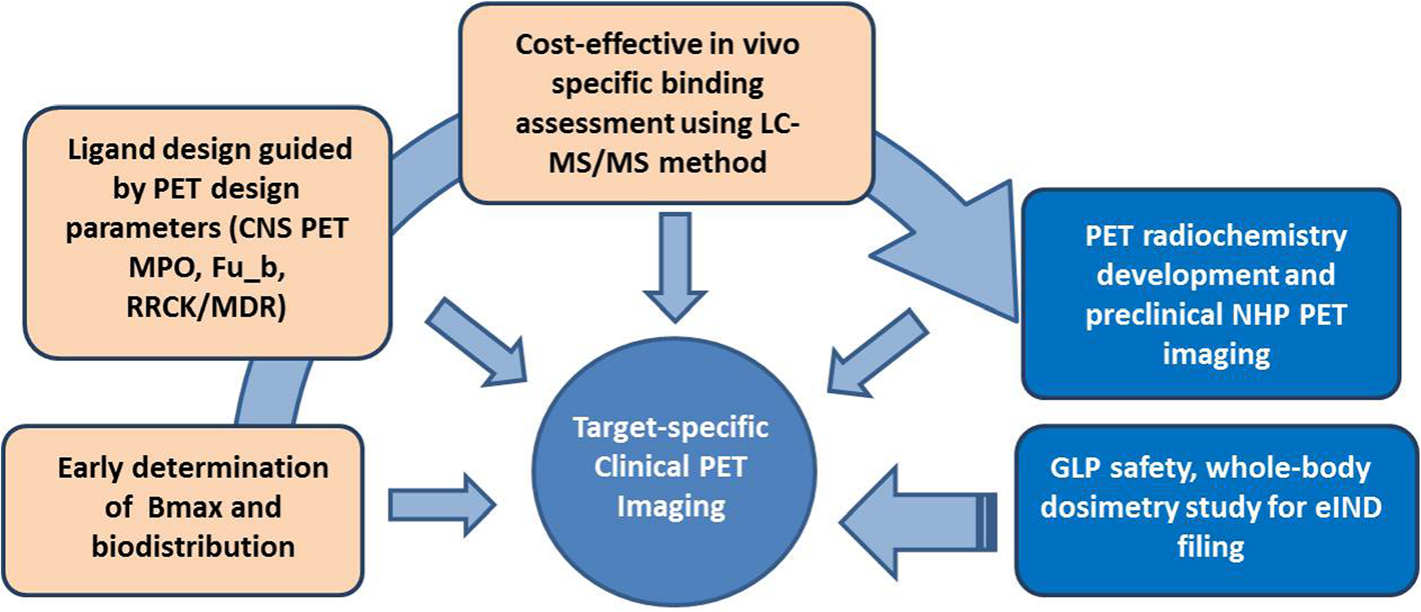
A cost-effective novel CNS PET ligand discovery process

The significant unmet medical need in CNS disorders calls for new strategies to improve the overall efficiency and success rate in drug discovery. PET, as a non-invasive translatable imaging technology, can be incorporated into various stages of the drug discovery process to provide valuable information for key preclinical and clinical decision-making. CNS PET ligand discovery efforts in the industry setting, however, are facing unique challenges associated with lead design and prioritization, and budget constraints. In an effort to expedite the discovery of novel PET ligands with a higher success rate and lower cost, we incorporated and integrated three strategies in the CNS PET ligand discovery process (Fig. 8), involving early determination of B_max_ and bio-distribution to inform PET viability and resource allocation, rational ligand design and lead prioritization guided by CNS PET design parameters, and a cost-effective in vivo specific binding assessment using a LC-MS/MS “cold tracer” method. Implementation of these strategies allowed a more focused CNS PET ligand discovery effort to identify the optimal ligand leads for PET imaging assessment, yielding a number of successful CNS PET ligands for novel neurotargets. Availability of these high quality PET ligands will undoubtedly broaden the use of PET imaging in the CNS drug discovery process, enable high quality preclinical and clinical studies, and ultimately result in the identification of novel treatment of neurological disorders of high unmet medical need.

## Abbreviations

AB: Apical-to-bisolateral
AD: Alzheimer’s disease
ADME: Absorption, distribution, metabolism and excretion
Aβ: β-amyloid
CNS: Central nervous system
Fu_b: Fraction unbound in brain
GLP: Good laboratory practice
HBD: Hydrogen bond donor
IV: Intra venous
KO: Knock-out
KOR: Kappa opioid receptor
LC-MS: Liquid chromatography-mass spectrometry
mGluR: Metabotropic glutamate receptor
MPO: Multi-parameter optimization
MW: Molecular weight
NHP: Non-human primate
NOP: Nociceptine opioid receptor
NSB: non-specific binding
P_app_: Apparent permeability coefficient
PD: Parkinson disease
PDE: Phosphodiesterase
PET: Positron emission tomography
PK: Pharmacokinetics
PO: Per oral
RRCK: Ralph-Russ canine kidney
SAR: Structure-activity relationship
TO: Target occupancy
tPSA: Topological polar surface area
WT: Wild-type
